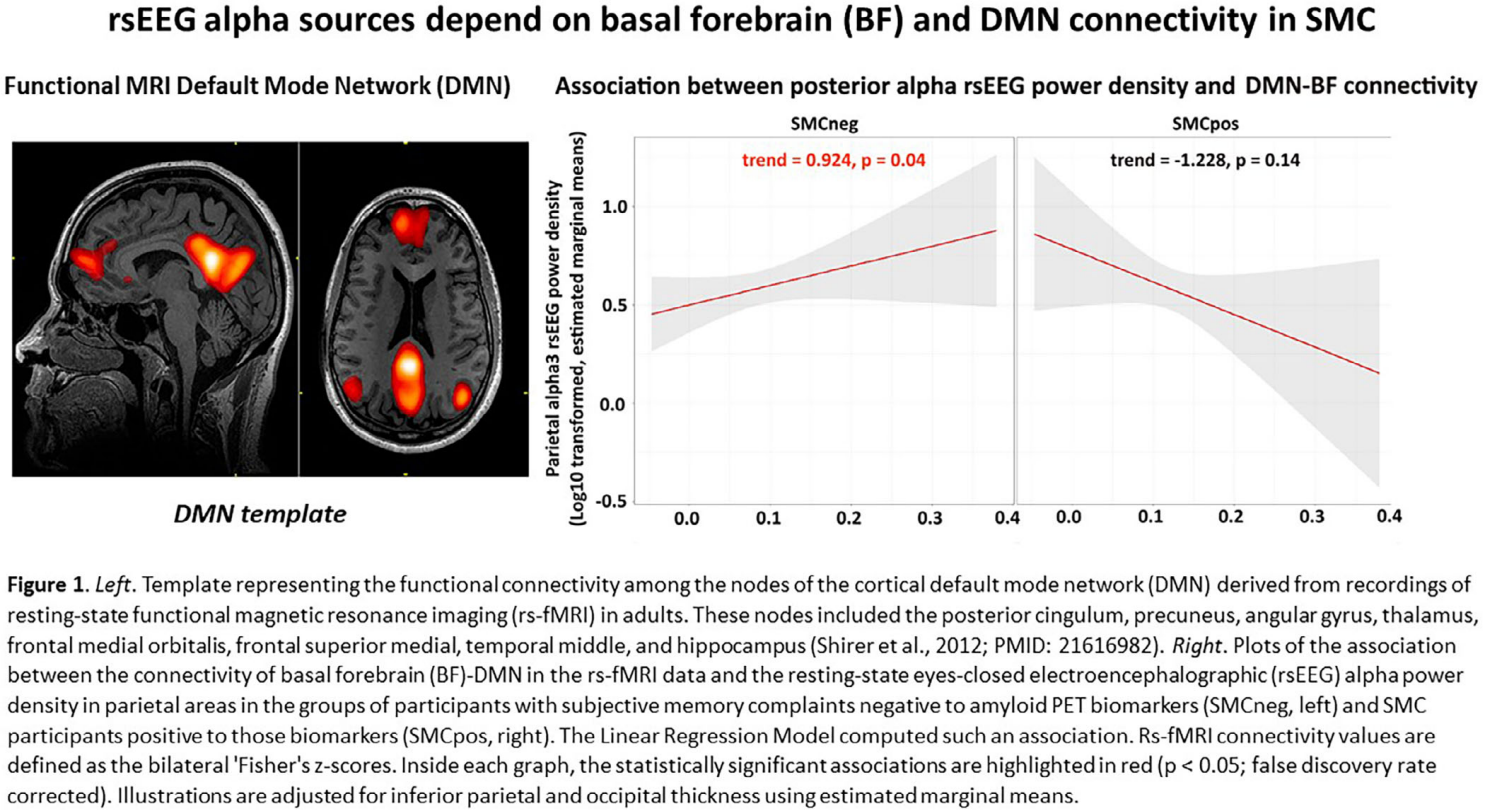# Default mode network and posterior resting‐state electroencephalographic alpha rhythms in Alzheimer’s disease patients

**DOI:** 10.1002/alz.084754

**Published:** 2025-01-09

**Authors:** Claudio Babiloni

**Affiliations:** ^1^ Department of Physiology and Pharmacology, Sapienza University of Rome, Rome Italy

## Abstract

**Background:**

Alzheimer’s disease dementia (ADD) is the most common neurodegenerative dementing disorder, explaining about 60‐70% of 50 million patients worldwide (www.who.int). Some previous studies in ADD patients showed significant neurodegenerative processes as revealed by reduced gray matter volume in the cerebral cortex, including the default mode network (DMN) cortical networks from structural magnetic resonance imaging (sMRI). Other studies in these patients reported a magnitude reduction in the resting‐state eyes‐closed electroencephalographic (rsEEG) alpha rhythms reflecting vigilance dysfunctions. Here, we tested the hypothesis of a significant relationship between these neurodegenerative and neurophysiological readouts, thus suggesting the sensitivity of rsEEG alpha rhythms to intrinsic AD processes.

**Methods:**

Clinical, sMRI, rs functional MRI, and rsEEG rhythms in demographic‐ and age‐matched ADD (N = 45), subjective memory complaint (SMC, N = 160), and healthy cognitively unimpaired (Nold, N = 40) persons were available from an international archive (www.pdwaves.eu). Individual alpha frequency peak was used to determine the alpha frequency band on a personal basis. The eLORETA freeware estimated rsEEG sources. Freeware platforms served to analyze MRI data.

**Results:**

The core results showed (1) lower DMN volumes and posterior rsEEG alpha rhythms in the ADD than Nold participants and (2) a positive association of the functional connectivity of basal forebrain‐DMN and the posterior rsEEG alpha rhythms in the SMC participants negative but not positive to amyloid PET biomarkers (Figure 1).

**Conclusions:**

A prominent relationship was observed between DMN and the posterior rsEEG alpha rhythms across physiological and AD‐related aging, suggesting the enrichment of the A‐T‐N Framework with "Pathophysiology" (P) EEG biomarkers.